# Septo-optic Dysplasia in a Patient With Increased Socioeconomic Needs: A Case Report

**DOI:** 10.7759/cureus.78479

**Published:** 2025-02-04

**Authors:** Aabid Mohiuddin, Adrianna Taweel, Wael Taha, Stephen Farrow

**Affiliations:** 1 Department of Internal Medicine, Detroit Medical Center/Wayne State University, Detroit, USA; 2 Department of Endocrinology, Detroit Medical Center/Wayne State University, Detroit, USA

**Keywords:** de morsier syndrome, diabetes insipidus, endocrinopathy, septo-optic dysplasia, social determinants of health

## Abstract

Septo-optic dysplasia (SOD) is a congenital disorder characterized by a triad of ophthalmologic, neurologic, and hypothalamic-pituitary developmental dysfunctions. This case describes a 25-year-old woman with SOD and multiple associated endocrinopathies, including diabetes insipidus, who presented to the hospital after running out of subcutaneous desmopressin that was adequately controlling her diabetes insipidus. Her family expressed concerns about the standard intranasal formulation due to a history of adverse effects. The endocrinology team developed a tailored treatment plan to address the family’s needs and limitations. This case highlights the role of endocrinologists in managing this rare disorder and the importance of tailored treatment plans for patients with complex needs.

## Introduction

Septo-optic dysplasia (SOD), also known as de Morsier syndrome, is a rare congenital disorder characterized by a triad of failures in ophthalmic, neurologic, and hypothalamic-pituitary development. Underdevelopment of the optic nerve, midline brain structures (including the corpus callosum), and pituitary gland leads to respective vision deficits, cognitive and motor impairments, and hormonal deficiencies. The most frequently seen hypothalamic-pituitary abnormalities are central hypothyroidism and growth hormone deficiency. Other endocrine abnormalities may occur, such as secondary, tertiary adrenal insufficiency or diabetes insipidus [1-4].

Incidence of SOD is rare, with an estimated 8 out of 100,000 live births affected. Life expectancy varies, as severity depends on the degree of disruptions across the different systems [5,6]. While the specific etiology of this condition is unclear, there is an association with intrauterine exposures including maternal diabetes, fetal alcohol syndrome, and maternal drug abuse [7,8]. As low socioeconomic status has been associated with higher rates of maternal alcohol consumption and drug abuse, it can therefore be inferred that children with SOD may face additional barriers to receiving appropriate care [9,10].

In this report, we describe the case of a patient with severe SOD born to a mother struggling with substance use. Unfortunately, the child experienced neglect as a result. The patient was eventually adopted by a family that has been attentive to her unique health needs. This case highlights the fundamental role of endocrinologists in the lifelong care of patients with this rare disorder.

## Case presentation

A 25-year-old woman with a history of Arnold-Chiari II malformation and SOD with resultant partial blindness, cognitive delay, and panhypopituitarism was brought to the hospital by her adoptive mother after exhausting their home medication supply and being unable to obtain refills due to an insurance issue. A comprehensive social history revealed that the patient was adopted from a group home with significant concern for neglect and malnourishment. The family reported that the patient was eight years old and weighed only 30 pounds at the time of adoption. Although a precise birth history was unavailable, documentation showed that the patient was born to a G3P3 19-year-old mother with a reported substance use history. The patient was admitted to the neonatal intensive care unit for three months after being born with the Arnold-Chiari malformation and associated hydrocephalus.

While the patient had vision deficits and cognitive delay, the more critical aspect of her long-term care was related to her endocrine care (specifically her panhypopituitarism and the repercussions of her hypothalamic-pituitary axis disruption). Her deficiencies in growth hormone and gonadotropic hormones were evident in her overall appearance, as she was short in stature and lacked secondary sexual characteristics. Her secondary hypothyroidism was treated with levothyroxine, while her secondary adrenal insufficiency was managed with hydrocortisone supplementation.

The most volatile element of her endocrine care was the management of her central diabetes insipidus. The patient’s history was notable for multiple hospitalizations due to seizures associated with relative hyponatremia. The family had previously managed her diabetes insipidus in her adolescent years with intranasal desmopressin at a dosage of 10 mcg twice daily. They noted that she was having more episodes of seizure-like activity, occurring monthly. After a discussion with her endocrinologist, she was switched to 2 mcg of subcutaneous desmopressin twice daily. On this dose, the seizures were controlled for several years until her current admission.

On this admission, the patient was brought in by her mother in anticipation of running out of desmopressin. She noted having to change her daughter’s diapers more frequently recently as she had begun rationing the doses due to her insurance no longer covering the subcutaneous formulation. The patient was alert, oriented at her baseline, and in no relative distress. She was small, appearing much younger than her stated age, and was nonverbal with a reserved disposition around the medical team. Her labs were significant for a marked hypernatremia of 160 mmol/L on admission, and the patient was admitted to the medical intensive care unit for monitoring.

Due to the high out-of-pocket cost of subcutaneous desmopressin, the endocrinology service was consulted for an alternative. A review of her records revealed that the patient was especially sensitive to fluctuations in her serum sodium levels. While on intranasal desmopressin at 10 mcg twice daily, she had frequent hospital admissions for seizures occurring in the setting of hyponatremia. We suspected that this dosage of desmopressin was supratherapeutic for the patient. The family was apprehensive about trialing the intranasal desmopressin again; however, we explained our rationale and recommended intranasal desmopressin at 10 mcg every 24 hours. The family was instructed to observe the moistness of her diapers as an approximate estimation of urine output: a dry diaper suggested excess retention indicative of supratherapeutic dosing, while frequent moist diapers implied insufficient dosage. This tailored recommendation was appropriate given the family’s high level of involvement and insight into her care. The patient was then discharged to care for her family with plans for prompt outpatient follow-up.

## Discussion

**Figure 1:**
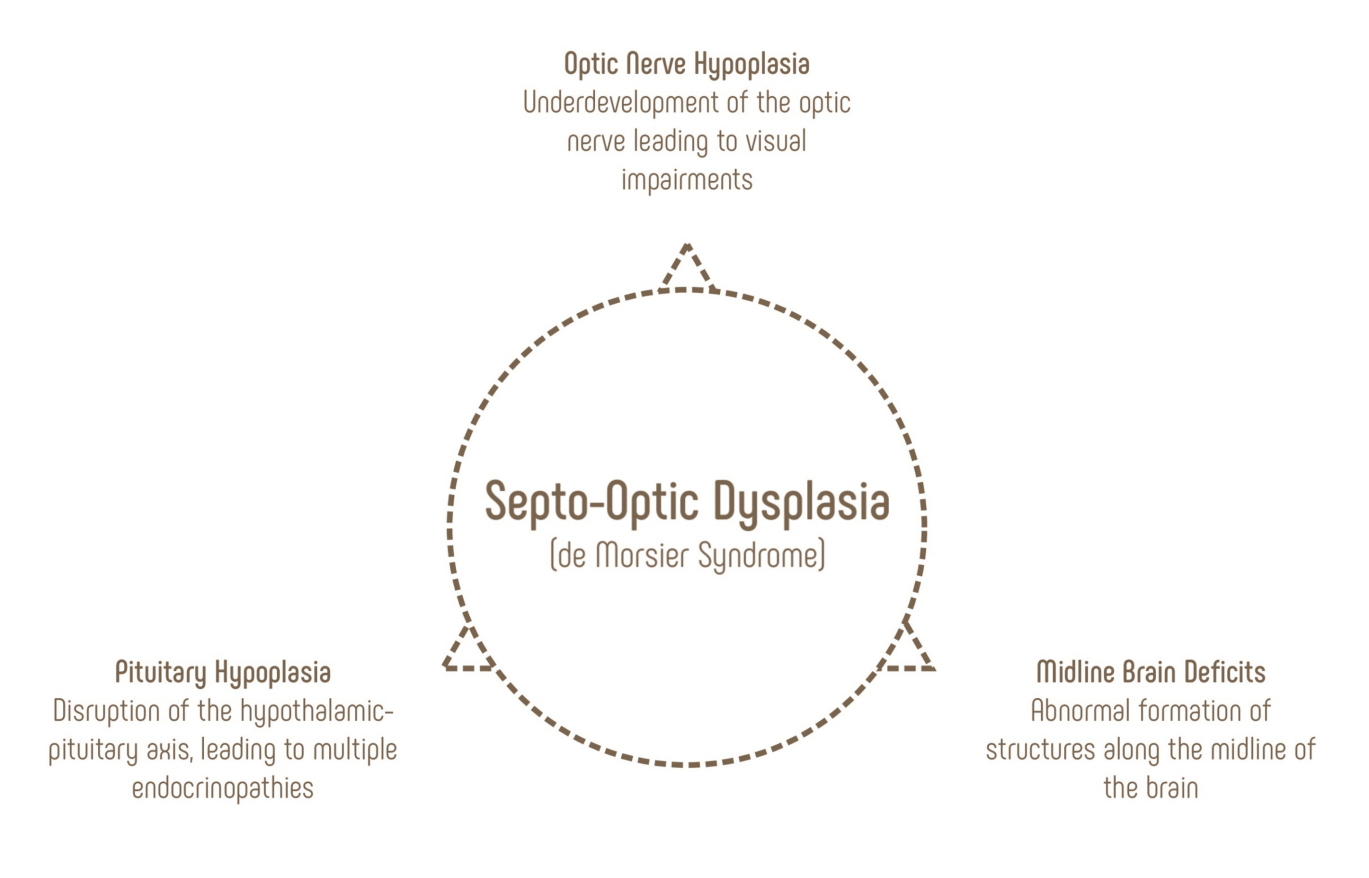
Septo-optic dysplasia is a rare disorder characterized by a triad of failures in ophthalmic, neurologic, and hypothalamic-pituitary development. Image credit: Authors' original creation.

SOD is exceptionally rare and occurs with a characteristic triad (Figure 1). Clinical evidence of combined ophthalmologic, developmental, and endocrine dysfunctions often triggers suspicion of the disorder, although the severity of the respective dysfunctions varies along a spectrum. Diagnosis is strengthened by magnetic resonance imaging (MRI) of the brain. In their retrospective study of patients with SOD, Cemeroglu et al. found that 96% of patients had MRI evidence of optic nerve hypoplasia, a multitude had MRI evidence of midline brain abnormalities including 85% of patients with absent/hypoplastic septum pellucidum, and 32% with absent/hypoplastic corpus callosum [4]. Pituitary hypoplasia is generally more difficult to distinguish on neuro-imaging and was only identified by MRI imaging in 9% of patients [4].

The characteristic triad helps differentiate SOD from similar conditions. For example, optic nerve hypoplasia is a leading cause of childhood blindness and can be associated with many disorders including fetal alcohol syndrome, maternal diabetes, and cerebral palsy. However, the presence of optic nerve hypoplasia with midline brain deficits and endocrine dysfunction is unique to SOD. Similarly, congenital brain malformations such as holoprosencephaly and agenesis of the corpus callosum are examples of isolated midline brain deficits occurring without the ophthalmologic and endocrine dysfunctions seen in SOD [7].

SOD requires multidisciplinary management to address its triad of dysfunctions. With regard to pituitary dysfunction, establishing care with pediatric endocrinology after diagnosis is crucial, with a transition to adult endocrinology when appropriate. A 2008 prospective study found that 80% of affected children had at least one endocrinopathy, including growth hormone deficiency (70%), hyperprolactinemia (62%), hypothyroidism (43%), adrenal insufficiency (27%), and diabetes insipidus (5%) [11].

While diabetes insipidus is a significant endocrine abnormality associated with SOD, its prevalence varies across studies. Masera et al. reported on the prevalence of diabetes insipidus in children with SOD and/or agenesis of the corpus callosum [12]. The study found that 9 out of 24 children had diabetes insipidus and that maintaining normal osmotic balance was particularly challenging in some cases, even with the introduction of vasopressin treatment [12]. Willnow et al. provided a detailed examination of the endocrine disorders associated with SOD in 18 patients, noting that all patients in their study exhibited some form of hormonal deficiency, with only one having central diabetes insipidus [3]. Hetman et al. reported a specific case of SOD where central diabetes insipidus was diagnosed [13]. A male infant, born at 39 weeks' gestation, faced challenges with breastfeeding due to a weakened sucking reflex. MRI at five months revealed SOD. Neurological examination identified axial hypotonia, delayed psychomotor development, and nystagmus. At three years, he developed polydipsia and polyuria, leading to a diagnosis of diabetes insipidus which was managed with desmopressin [13].

Due to the profound endocrinopathies associated with this condition, it is vital for endocrinologists to routinely follow these patients. Our case represents a severe occurrence of SOD, with profound deficiencies noted across all elements of the hypothalamic-pituitary axis. Her central diabetes insipidus, the rarest of the associated endocrinopathies, was complicated by intolerance of the intranasal desmopressin formulation as she was prone to seizures when on this formulation. However, antidiuretic hormone is not secreted cyclically; therefore, timed dosing of desmopressin can often lead to supra- or sub-therapeutic levels based on the patient’s overall hydration status. Through an understanding of diabetes insipidus management, as well as a creative approach to dosing (i.e., judging appropriateness via moistness of the patient’s diaper), the endocrinology service was able to provide an effective and attainable treatment regimen to the family.

## Conclusions

Effective management of SOD necessitates a multidisciplinary approach, with attentive care by endocrine specialists being crucial to address the endocrinopathies. Furthermore, as the sequelae of SOD begin in early childhood and continue into adulthood, an appropriate transition in care from pediatric to adult endocrinology is required. The necessary lifelong care can levy severe financial and emotional burdens on patients’ families; clinicians should be aware of this reality and advise clinical treatment plans that account for those potential limitations. Ultimately, this case highlights a rare, severe condition while also demonstrating how physicians can take innovative approaches to providing effective care for financially constrained patients without compromising standards.
